# Increased Genetic Variance of BMI with a Higher Prevalence of Obesity

**DOI:** 10.1371/journal.pone.0020816

**Published:** 2011-06-29

**Authors:** Benjamin Rokholm, Karri Silventoinen, Lars Ängquist, Axel Skytthe, Kirsten Ohm Kyvik, Thorkild I. A. Sørensen

**Affiliations:** 1 Institute of Preventive Medicine, Copenhagen University Hospital, Centre for Health and Society, Copenhagen, Denmark; 2 Population Research Unit, Department of Social Research, University of Helsinki, Helsinki, Finland; 3 The Danish Twin Registry, Institute of Public Health, University of Southern Denmark, Odense, Denmark; University of Florence, Italy

## Abstract

**Background and objectives:**

There is no doubt that the dramatic worldwide increase in obesity prevalence is due to changes in environmental factors. However, twin studies suggest that genetic differences are responsible for the major part of the variation in body mass index (BMI) and other measures of body fatness within populations. Several recent studies suggest that the genetic effects on adiposity may be stronger when combined with presumed risk factors for obesity. We tested the hypothesis that a higher prevalence of obesity and overweight and a higher BMI mean is associated with a larger genetic variation in BMI.

**Methods:**

The data consisted of self-reported height and weight from two Danish twin surveys in 1994 and 2002. A total of 15,017 monozygotic and dizygotic twin pairs were divided into subgroups by year of birth (from 1931 through 1982) and sex. The genetic and environmental variance components of BMI were calculated for each subgroup using the classical twin design. Likewise, the prevalence of obesity, prevalence of overweight and the mean of the BMI distribution was calculated for each subgroup and tested as explanatory variables in a random effects meta-regression model with the square root of the additive genetic variance (equal to the standard deviation) as the dependent variable.

**Results:**

The size of additive genetic variation was positively and significantly associated with obesity prevalence (p = 0.001) and the mean of the BMI distribution (p = 0.015). The association with prevalence of overweight was positive but not statistically significant (p = 0.177).

**Conclusion:**

The results suggest that the genetic variation in BMI increases as the prevalence of obesity, prevalence of overweight and the BMI mean increases. The findings suggest that the genes related to body fatness are expressed more aggressively under the influence of an obesity-promoting environment.

## Introduction

The prevalence of obesity has increased at epidemic rates in most parts of the world in the last decades, and in the USA recent data show that more than a third of all men and women are now obese [1], [2]. There is no doubt that environmental factors have initiated the epidemic, since the gene-pool in the population changes at a rate that is much too slow to explain the observed pattern. However with heritability of body mass index (BMI) in the range of 0.5 to 0.8, twin studies suggest that genetic differences between individuals are responsible for the majority of variation in body fatness within populations [3], [4]. In the last years genome wide association studies have aimed at identifying the genetic loci responsible for the high heritability estimates. The results are, however, somewhat discouraging since the accumulated influence of the genetic loci identified to date account for only slightly over two percent of the total genetic variation in BMI [5]. It has been suggested that part of the discrepancy between heritability and the genetic variation from currently identified genes could be attributable to interaction effects between genetic loci, i.e. epistatic effects, or interaction between genes and the environment. The latter implies that the effect of genes depend upon the environmental exposure and vice versa. This is modelled in twin and family studies as part of genetic variation but not found in genome wide association studies, which usually only focus on the main effects of candidate genes [6], [7]. Until now the focus has primarily been on physical activity as a potential modifier of the genetic effects on adiposity. Twin studies conducted in several populations have found lower heritability of obesity in physically more active individuals [8]–[10]. Similarly on a molecular genetic level, several studies have recently found that physical activity attenuates the effect of the fat mass and obesity associated (FTO) gene and other genetic loci that are associated with body fatness [11]–[14]. In addition, fat and carbohydrate intake has been found to interact with the FTO gene on BMI [15]. These results all suggest that genetic effects on adiposity are modifiable by environmental influences. However, the research is still at an early stage and it is possible that the discovered interactions apply to other genetic loci and environmental factors than physical activity, fat and carbohydrate intake. On this background we tested the more general hypothesis that the genetic variance component of BMI is higher in populations with a higher prevalence of obesity, a higher prevalence of overweight and a higher mean BMI. If the hypothesis is confirmed it may imply that the obesity-promoting environment modifies the effect of genes related to adiposity.

## Methods

### Subjects

The study population originates from The Danish Twin Registry (DTR), which contains information on virtually all twins born in Denmark since 1870 [16]. In 1994 and 2002, two questionnaire surveys were conducted with twins from the DTR born between 1953 and 1982, and 1931 and 1982, respectively. In both questionnaires the participants answered questions regarding health related behavior and outcomes. In the current study we used information on height and weight together with year of birth, gender, and zygosity, which have been assessed in previous questionnaire surveys [17]. With an overall misclassification rate of only 4%, questionnaire based zygosity assessment is considered to be a valid classification method for most purposes [17]. BMI was used as a measure of body fatness and was calculated from self reported height and weight (BMI; weight in kilograms divided by squared height in metres [kg/m^2^]).

**Figure 1:**
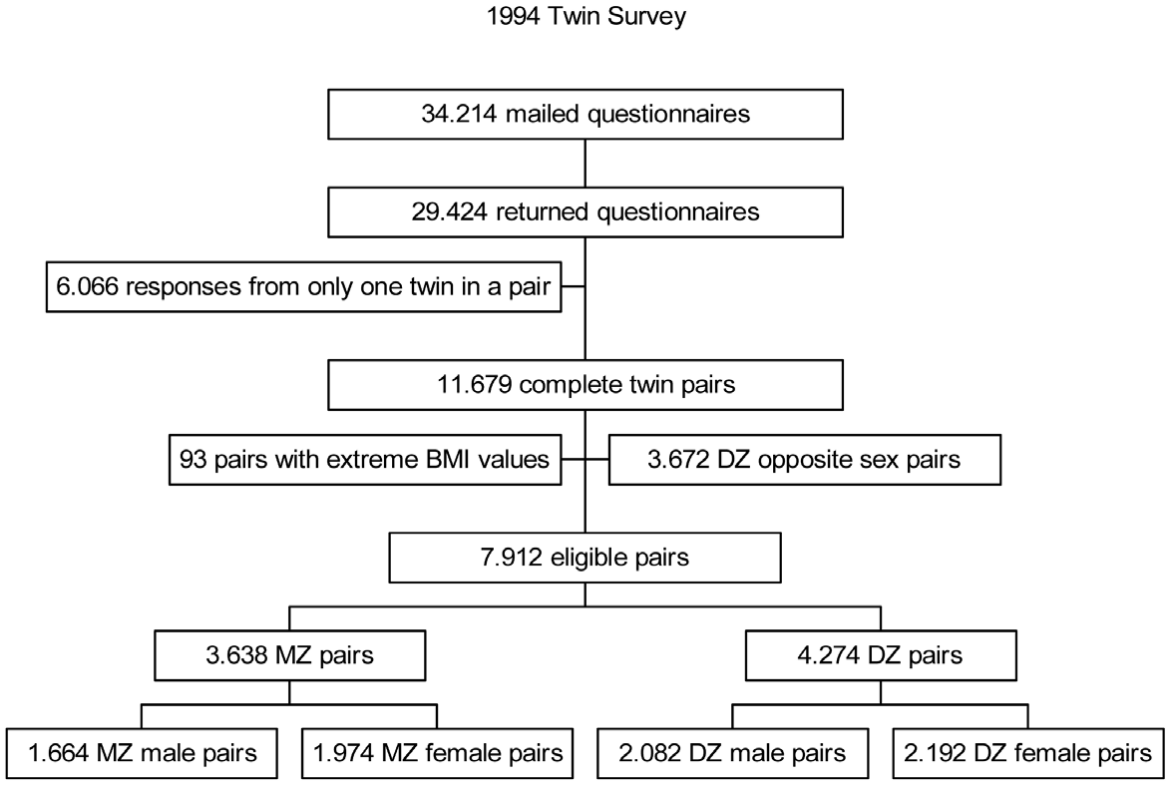
Flowchart for selection of twins from the 1994 survey. The flowchart shows how eligible twin pairs were selected from the twin survey conducted in 1994. From the returned questionnaire we excluded twin pairs with incomplete information on one of the twins in a pair, opposite sex twin pairs and twin pairs with extreme BMI values.

**Figure 2:**
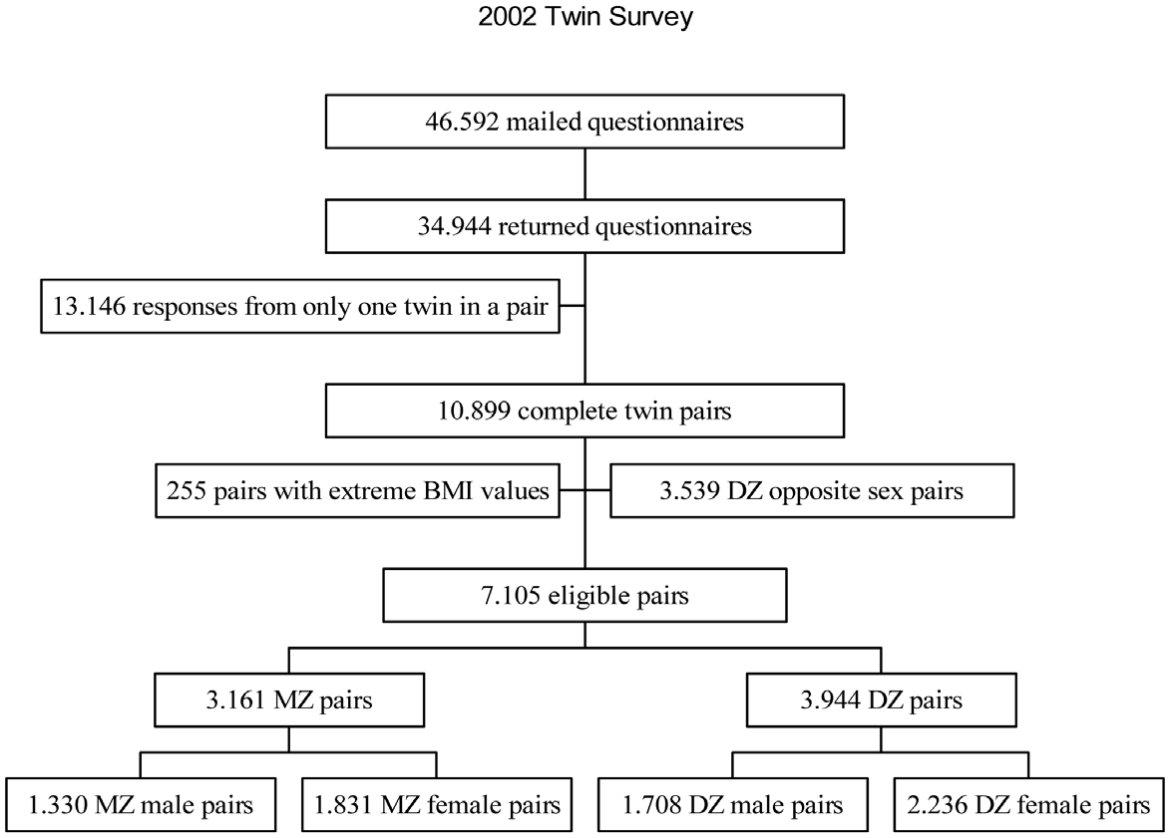
Flowchart for selection of twins from the 2002 survey. The flowchart shows how eligible twin pairs were selected from the twin survey conducted in 2002. From the returned questionnaire we excluded twin pairs with incomplete information on one of the twins in a pair, opposite sex twin pairs and twin pairs with extreme BMI values.

A total of 29,424 individuals responded to the 1994 survey, corresponding to a response rate of 86%. In 11,679 twin pairs information on height and weight was found in the responses from both twins in a pair. Dizygotic opposite-sexed twin pairs (3,674 pairs) were not used in the calculation of the variance components, and additional 93 pairs were excluded due to extreme BMI values (BMI<15 kg/m^2^ or BMI>50 kg/m^2^). Thus, a total of 7,912 twin pairs were available for the analysis, including 1,664 MZ male, 2,082 DZ male, 1,974 MZ female and 2,192 DZ female twin pairs (Figure 1). The 2002 survey resulted in 34,944 individual responses, yielding a response rate of 75%. From the individual responses 10,899 complete twin pairs with information on height and weight were identified. Of these, 3,539 pairs were opposite sex dizygotic twins and were excluded from the analysis. From the remaining 7,360 twin pairs we excluded 255 pairs with extreme BMI values (BMI<15 kg/m^2^ or BMI>50 kg/m^2^) resulting in 7,105 twin pairs for the analysis including 1,330 MZ male, 1,708 DZ male, 1,831 MZ female and 2,236 DZ female twin pairs (Figure 2).

### Statistical analysis

The data within each defined subgroup (sex-birth year strata) were analysed through quantitative genetic modelling, in which the total phenotypic variation of a trait is divided into variation due to environmental factors and variation caused by genetic differences between individuals. The disentangling of genetic and environmental variation is made possible through structural equation modelling of co-variation within pairs of different types of family relations. The relations included in the modelling are genetically informative if they differ in the degree of genetic or environmental similarity [18]. In the case of monozygotic and dizygotic twin pairs, the genetic and environmental variation can be disentangled since monozygotic twins have the same gene sequence, while dizygotic twins share, on average, 50% of their segregating genes.

The genetic variance can further be decomposed into parts due to additive effects of alleles at multiple loci (A) and due to dominance effects (D) at multiple loci, respectively. Epistatic effects refer to interaction between genes at different loci and will usually be modelled as part of dominant genetic effects. However in the case of linked loci, epistatic effects will be modelled as additive genetic effects since the genes segregate together and form a unit. The environmental variance can be divided into factors shared by co-twins (C) and factors that are unique to each twin individual including also any measurement error (E). Hence, total phenotypic variation can be decomposed into four components: the additive genetic (A), dominant genetic (D), common environmental (C), and specific environmental (E) variance component. Since we only have data on twins reared together, the D and C components cannot be estimated simultaneously and thus the model fit of the ACE and ADE models were compared [18]. In practice the components are calculated using the Mx statistical software, version 1.7.03, using the raw data option, which allows the implementation of twins without information on their co-twins [19]. Mx derives structural equation models from twin and family data and calculates the variance components through maximum likelihood estimation. Furthermore, the significance of each variance component was tested and the most parsimonious model was selected. In the current analysis the AE model was found to give the best fit and was hence used throughout.

As noted above, the study population was stratified by sex and birth year giving a total of 164 subgroups. The division into birth cohorts ensures that the gene pool is similar across subgroups and at the same time allows variation in the explanatory variables. Hence, any differences in variance components between subgroups, when controlling for age and sex, are caused by environmental factors and not by differences in genetic background. The variance components were subsequently calculated for each subgroup. To simplify the analyses and interpretation of the variance components estimates, opposite sex dizygotic twin pairs were excluded from the analysis.

The obesity prevalence, overweight prevalence and mean BMI were calculated for each subgroup defined by sex and year of birth (these statistics were calculated from the whole twin population including opposite sex dizygotic twin pairs to get more precise estimates). The underlying assumption is that an obesity promoting environment will result in a higher prevalence of obesity and overweight and a higher mean BMI [20], [21]. In accordance with The World Health Organisation guidelines, overweight and obesity among adults were defined as a BMI> = 25 and 30 kg/m^2^, respectively [22]. For adolescents, internationally standardised age and sex specific cut-points were used [23].

The prevalence of obesity and overweight and the BMI mean were tested as explanatory variables for the (square-root of the) additive genetic variance (AGV), using random effects meta-regression modelling (REMR) [24]. REMR is a statistical approach in which populations, or subgroups of a larger population, are the unit of analysis in a regression analysis. The outcome variable, in this case AGV, is assessed for each subgroup and various characteristics of the subgroups are treated as explanatory variables for the inter population differences in the outcome. Each subgroup is weighed by a function of the inverse of the estimated variance of the corresponding estimate (more explicitly, the function corresponds to the inverse of the sum of this variance and the estimated study-heterogeneity parameter) [25]. This implies that subgroups with larger sample sizes are usually given more weight in the analysis, since standard errors and sample sizes in most cases will be quite strongly negatively correlated (even on the between-study level). In other words, studies with small sample sizes will generally be given a low influence in the meta-analyses. In practice we used the additive genetic standard deviation (AGSD) as dependent variable, since the Mx software only reports standard errors of the additive genetic standard deviation and not of the AGV. However, since the standard deviation is simply the square root of the AGV, an increase in the standard deviation implies an increase in the AGV (i.e. the relation is one-to-one and monotonically increasing, which indicates consistency of results over approaches). The meta-regression analysis was conducted using the “Metareg” procedure in the STATA statistical software [26]. The effects of the proxy variables were controlled for age, sex and survey year. Interaction effects between proxy variables and age and were also tested.

## Results

The analyses included 6,799 monozygotic and 8,218 dizygotic eligible twin pairs. Summary statistics of BMI by survey, zygosity and sex is reported in Table 1.

**Table 1:** Summary statistics of twin data.

Survey 1 (1994)				
	MZ twins		DZ twins (same sex)	
	Male	Female	Male	Female
No. twin pairs	1,664	1,974	2,082	2,192
BMI Mean	22.48	21.29	22.85	21.81
BMI Variance	9.77	9.83	8.40	9.04
Total no. twin pairs	3,638		4,274	

Survey 2 (2002)				
	MZ twins		DZ twins (same sex)	
	Male	Female	Male	Female
No. twin pairs	1,330	1,831	1,708	2,236
BMI Mean	25.15	23.24	25.28	23.71
BMI Variance	9.30	12.82	7.55	11.08
Total no. twin pairs	3,161		3,944	

The explanatory variables and AGV are reported for each subgroup in Table S1. In accordance with the literature the estimates of heritability ranged from about 50% to 90%, indicating that genetic factors explain most of the variation in BMI in all subgroups. The prevalence of overweight and obesity ranged from 2.3% to 64.5% and 0% to 15.6%, respectively. Mean BMI ranged from 17.8 to 26.5 kg/m^2^.

**Figure 3:**
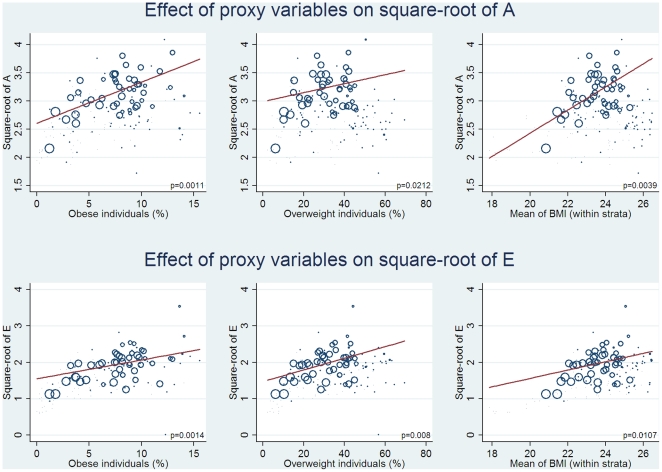
Regression models for the A (additive genetic) and E (unique environmental) component. The (square root of the) additive genetic variance and unique environmental variation is plotted against each of the proxy variables obesity prevalence, overweight prevalence and the mean of the BMI distribution. Each circle represents a subgroup. The size of the circle is inversely proportionate to the standard error of AGV for each subgroup. The regression line shows the best fit with larger circles given more weight. The blue punctured and red regression line represents the stratified analyses for males and females, respectively.

Table 2 shows parameter estimates and corresponding p-values for the REMR-models including the explanatory variables controlled for sex, age and survey year for both the additive genetic and unique environmental standard deviation. Both the additive genetic and unique environmental standard deviation, and thus the genetic and environmental variance components, was positively and statistically significantly associated with prevalence of obesity and the mean of the BMI distribution. The association with prevalence of overweight was positive, but not statistically significant. The genetic standard deviation increased roughly 0.1 units with every one percentage point increase in the prevalence of obesity. The average genetic standard deviation was around 3 units for the whole population. Thus, a one percentage point increase in the prevalence of obesity is associated with an increase in the genetic standard deviation of about 0.1/3 = 3.3%. Figure 3 plots the estimates of the additive genetic and the unique environmental standard deviation against prevalence of obesity, prevalence of overweight and the mean of the BMI distribution. Each circle represents a subgroup with the size inversely proportional to the corresponding random effects meta-analysis weight (defined as described above).

**Table 2:** Results from Random Effects Meta-regression Modelling.

	A (standard deviation)		E (standard deviation)	
	Parameter est.	P-value	Parameter est.	P-value
Obesity prevalence				
Obesity (%)	0.095	0.001	0.081	0.012
Sex	−0.223	0.839	−0,231	0.850
Age	−0,009	0.065	−0,002	0.747
Sex*Obesity	−0.035	0.770	−0.023	0.862
Survey	−0.228	0.114	−0.068	0.662
Overweight prevalence				
Overweight (%)	0.023	0.177	0.032	0.064
Sex	−0.217	0.873	−0.539	0.720
Age	−0.011	0.288	−0.010	0.346
Sex*Overweight	−0.012	0.692	−0.007	0.841
Survey	−0.290	0.125	−0.025	0.894
Mean BMI				
Mean (BMI)	0.376	0.015	0.323	0.050
Sex	2.729	0.749	−0.225	0.983
Age	−0.017	0.050	−0.008	0.379
Sex*Mean	−0.150	0.660	−0.026	0.950
Survey	−0.102	0.603	0.037	0.859

The standard deviation of the additive genetic component and unique environmental component are modelled as dependent variables. Parameter estimates and p-values are reported for the explanatory variables, which are listed in the first column. Three models were tested - one for each of the three main variables of interest: prevalence of obesity, prevalence of overweight and mean BMI.

A large proportion of twins from the first survey were also present in the second survey (about 72%). This is a potential problem in the statistical modelling since not all observations are independent. Hence, in order to determine whether the overlap would bias the results, we carried out the same analyses excluding the overlap between the two surveys. We found that the results were not statistically significantly different when overlaps were excluded.

## Discussion

We found that both the additive genetic and unique environmental standard deviations were positively associated with prevalence of obesity and the mean of the BMI distribution. The association with overweight, although not statistically significant, was also positive. Since the gene pool can be assumed to be comparable across subgroups, the findings suggest that the environmental influence, causing a higher prevalence of obesity and a higher BMI mean, increases both the genetic and environmental variance of BMI. Hence, an obesity-promoting environment is possibly modifying the genetic variance in BMI. As will be argued later, the current findings may be comparable with recent findings on specific environmental modifiers of genetic variance.

Although not explicitly a part of the hypothesis, it is worth noting that also the environmental variance was higher in subgroups with a higher prevalence of obesity and mean BMI (Figure 3). This suggests that in a more obesity-promoting environment there is also a larger degree of inter-individual heterogeneity in the exposure to the environmental factors related to obesity. In terms of the development of the obesity epidemic, the results suggest that the inter-individual differences in the degree of exposure to the obesity-promoting environment increases over time. In other words, the environmental influence grows stronger, but only for a limited part of the population.

Previous studies have found higher AGV of BMI and waist circumference among individuals with a low, compared to high, level of physical activity [8], [27], [28]. Assuming that physical activity is associated with the obesity-promoting environment, these results are in concordance with the current study, i.e., the obesity-promoting environment induces a higher genetic variance of BMI. Since the AGV comprises the sum of all additive allelic effects influencing phenotypic variance [18], increase in AGV may be interpreted as an increase in the effect of one or several genetic loci. Interestingly, previous studies have found the effect of the FTO gene, which is associated with body fatness, to be higher among individuals with a low, compared to high, level of physical activity [11]–[14]. However, the FTO gene explains a very small part of the total genetic variance of BMI and the FTO is unlikely to alone explain the increase observed in AGV. Thus it is possible that effects of additional loci are modified under the influence of an obesity-promoting environment. This hypothesis is supported by a recent study in which 12 SNPs in obesity-susceptibility loci of 20,430 individuals were genotyped [14]. Each additional BMI-increasing allele was associated with 0.15 kg/m^2^ increase in BMI. This association was significantly more pronounced in physically inactive (0.21 kg/m^2^) than in physically active (0.13 kg/m^2^) individuals. Environmental influences other than physical activity are possibly contributing to changes in the expression of genes related to body fatness as well. For example, fat and carbohydrate, but not protein, intake was found to interact with the effect of FTO-gene on BMI [8], [15].

The mechanisms involved in the increase in AGV could occur on at least two separate levels. One possibility is that the changes in genetic variation reflect changes in gene regulation at a molecular genetic level. Alternatively the associations could be the result of more distal environmental influences on gene expression. For example, conceive of a genotype that is associated with high body fatness due to increased appetite. If the individual carrying the genotype lives in an environment where food is limited, the particular genotype will not be allowed full expression on body fatness. Nevertheless, changes in environmental conditions, allowing easier access to high energy-dense foods, would lead to an increased expression of the particular genotype on body fatness. This, in turn, could result in an increase in AGV of body fatness. In this light, the current results could reflect differences in environmental conditions across birth cohorts, enabling or restricting the expression of one or several genes associated with BMI.

Gene-environment correlation (rGE) is a phenomenon closely related to the possible distal regulations of gene-expression. rGE refers to a phenomenon in which the environmental exposure of an individual is related to his genotype. For example, a certain personality trait, which is genetically influenced, may result in an individual preference for an urban environment. If the urban environment is more obesogenic than the rural, then this rGE would not be distinguishable from other types of genetic effects on adiposity. If the urban environment becomes increasingly obesogenic with time this could increase the genetic variance and hence explain the current results. Finally it should be noted that the mechanisms could occur at both levels and in theory act synergistically and perhaps even cancel each other out.

The value of the current study has both a practical and theoretical dimension. We will consider the practical value first. Since the additive genetic variance component is an aggregate of the effect of all genetic loci, the regression models can potentially be used as a tool to optimize the search for adiposity related genes. It is plausible that by selecting populations with model parameters giving the highest predicted additive genetic variance, we are improving our chances of locating genes related to body fatness. Furthermore, it is possible that the increase in additive genetic variance across different levels of obesity prevalence reflects the initiation of new genes, not active at lower exposure levels. In this case the model could improve not only efficiency, but also the possibility of locating new loci not previously discovered. It can therefore be argued that, based on the current findings, the genome wide association studies should be carried out in populations with a high prevalence of obesity, high prevalence of overweight and a high mean BMI. In addition, it is worth noting that inconsistent results from genome wide association studies on obesity may, in part, result from inter-population differences in the exposure to the obesity-promoting environment.

Knowledge on environmental modification of gene expression may also, in a longer term, be used in prevention or treatment of obesity. If specific modifiable environmental factors are associated with the effect of particular genetic variants, the results could possibly be used as a measure of prevention or treatment in an individualised form. It is, however, possible that gene-environment interaction studies, on a molecular level, will show limited success in explaining the bulk of the variation, as the case has been with simple genome-wide association studies. An alternative approach would be to imply knowledge of gene-environment interaction on a population-based level. If we, through twin and family studies, are able to identify modifiable environmental factors, which have a considerable impact on genetic variance of adiposity, we can use the results in population level prevention without knowledge of the effect on particular genes. However, in this case the approach to obesity prevention will, in practice, not be different from the current public health efforts, unless new environmental modifiers, not previously considered risk factors of obesity, are discovered.

The theoretical value of this work lies in the realisation that the expression of adiposity-related genes is highly dependent on the environmental context. The increase in genetic variance was estimated to around 3.3% for every one percentage point increase in prevalence of obesity. This translates into a 33.3% increase with a 10 percentage point increase in the prevalence of obesity, which is seen in many countries. Hence, based on the current statistical modelling it is possible that the genetic standard deviation has increased considerably in many places. This implies that gene-environment interaction may be responsible for a large part of the genetic variance and that the genetic architecture of obesity should not be considered independent from the environmental context. Hence, there may be a substantial benefit in, to a further extent, incorporating gene-environment interaction into both the molecular and the quantitative genetic modelling of the genetic architecture of obesity.

The current study is based on data from two cross-sectional surveys. If the changes in prevalence of obesity were only a product of calendar time, there would be no variation in exposure within subgroups in the same survey. However, Olsen et al. showed that the Danish obesity epidemic is most likely more directly associated with birth cohorts than with calendar time [29]. Hence by forming strata based on birth-year, we ensure a sufficient variation in the explanatory variables. The inter-groups variation is also apparent from the relatively large differences between the lowest and highest values of the explanatory variables. Additionally, the wide age-range - as observed primarily within surveys but also between surveys - contributes to this variation.

An important strength in the current study design is that the gene pool across subgroups can be expected to be the same, since the genetic architecture is unrelated to year of birth. This implies that any differences in genetic variance are caused by differences in environmental influence and not by systematic differences in genetic material between subgroups. In other words, the discovered association between the explanatory variables and the genetic variance component may be attributed to environmental modification of the effect of genes related to BMI.

The current study is also subject to limitations. After excluding incomplete twin pairs, opposite sex twin pairs and twin pairs with extreme BMI values only 47% of the individuals from the total sample are included in the analyses. We found the BMI variation to be slightly higher among opposite sex twin pairs. Thus, theoretically, the results may be biased by selection of individuals who are different from the total original sample. However, our main interest is in the genetic variance. Biologically there is no reason to assume that various types of siblings differ in terms of their gene pool, why this limitation is unlikely to be critical in the current study. Height and weight was self-reported which is a less accurate method to assess BMI than standardized measurements. Since measurement errors are modelled as unique environmental variance it is likely that the heritability of BMI would be underestimated. However, when estimating the AGV we are using the raw estimate, which, in contrast to heritability, is not dependent upon the environmental variance component. Another issue is that the obesity-promoting environment was measured indirectly through the obesity prevalence, overweight prevalence and mean BMI. Thus, the results do not clarify which specific factors in the environment induce the observed changes. Although previous studies give evidence of physical activity as a modifier, it is possible that other factors are involved as well. Likewise, the study is limited to addressing general changes in gene expression and more work is needed on a molecular genetic level in order to elucidate the specific genetic units involved. Although the study was designed to eliminate confounding from differences in the gene-pool between strata (by stratifying by birth year), residual confounding can never be completely ruled out in observational studies. For example, age may confound the results if the particular parameterization of the age-effect does not capture the relationship between age and the AGV. Another limitation is that analyses were carried out in Denmark where the prevalence of obesity is relatively low compared to, for example, the USA [29]. It would be interesting to carry out the same analyses in more obese populations to see whether the genetic variance continues to increase beyond the limits investigated here.

In summary, our study shows that the additive genetic and environmental variance is positively associated with prevalence of obesity, prevalence of overweight and the mean of the BMI distribution, although not statistically significant for overweight. The results suggest that the obesity-promoting environment enhances the effect of genes related to body fatness. The findings may be related to previous studies showing a higher genetic variance and a larger effect of the FTO-gene among individuals with a low, compared to high, physical activity level. The association could reflect changes in genetic regulation at a molecular genetic level or be the result of more distal environmental restrictions on gene expression. In near term the results may be utilized in the search for new candidate genes by defining study populations that give the highest predicted genetic variance. In a longer term the research into gene-environment interaction may have a potential in future obesity prevention, either as part of an individualized or a population-based approach.

## Supporting Information

Table S1 **Subgroups used in meta-regression analyses.** Subgroups defined by stratifying the twin sample by survey, sex and year of birth. For each subgroup the summary statistics (obesity prevalence, overweight prevalence and BMI mean), additive genetic variance (AGV) and number of twin pairs (N) is reported.
(DOC)
