# Supplementary material for: Increased Genetic Variance of BMI with a Higher Prevalence of Obesity
Source: PLoS One. 2011 Jun 29;6(6):e20816. doi: 10.1371/journal.pone.0020816 (PMC3126806; doi:10.1371/journal.pone.0020816)
Supplement: Table S1 — Subgroups used in meta-regression analyses. Subgroups defined by stratifying the twin sample by survey, sex and year of birth. For each subgroup the summary statistics (obesity prevalence, overweight prevalence and BMI mean), additive genetic variance (AGV) and number of twin pairs (N) is reported. (DOC) [file pone.0020816.s001.doc]

Table S1

| Survey year | Birth year | Sex | Obesity (%) | Overweight (%) | Mean | AGV | N |
| --- | --- | --- | --- | --- | --- | --- | --- |
| 2002 | 31 | Male | 9.9 | 53.9 | 25.7 | 8.4 | 37 |
| 2002 | 32 | Male | 9.6 | 56.7 | 25.7 | 2.9 | 42 |
| 2002 | 33 | Male | 9.3 | 51.6 | 25.5 | 6.3 | 41 |
| 2002 | 34 | Male | 13.9 | 59.5 | 26.3 | 5.8 | 39 |
| 2002 | 35 | Male | 9.4 | 54.0 | 25.9 | 4.8 | 54 |
| 2002 | 36 | Male | 15.6 | 63.3 | 26.5 | 7.7 | 48 |
| 2002 | 37 | Male | 12.4 | 54.7 | 26.2 | 10.3 | 49 |
| 2002 | 38 | Male | 13.7 | 60.6 | 25.9 | 5.3 | 50 |
| 2002 | 39 | Male | 13.2 | 64.5 | 26.3 | 9.1 | 58 |
| 2002 | 40 | Male | 10.6 | 69.6 | 26.3 | 7.1 | 63 |
| 2002 | 41 | Male | 12.3 | 56.8 | 26.0 | 9.1 | 62 |
| 2002 | 42 | Male | 14.0 | 60.3 | 26.3 | 6.2 | 72 |
| 2002 | 43 | Male | 9.8 | 62.3 | 26.4 | 6.8 | 82 |
| 2002 | 44 | Male | 10.8 | 58.5 | 26.1 | 6.2 | 76 |
| 2002 | 45 | Male | 10.6 | 60.3 | 26.4 | 6.9 | 93 |
| 2002 | 46 | Male | 14.7 | 54.9 | 26.0 | 7.8 | 95 |
| 2002 | 47 | Male | 10.9 | 54.3 | 25.9 | 7.3 | 102 |
| 2002 | 48 | Male | 11.0 | 57.7 | 26.0 | 6.8 | 99 |
| 2002 | 49 | Male | 6.5 | 54.2 | 25.7 | 6.2 | 89 |
| 2002 | 50 | Male | 8.3 | 49.1 | 25.6 | 7.3 | 78 |
| 2002 | 51 | Male | 9.3 | 51.3 | 25.5 | 8.5 | 75 |
| 2002 | 52 | Male | 9.8 | 46.6 | 25.4 | 7.4 | 80 |
| 2002 | 53 | Male | 9.5 | 50.4 | 25.6 | 6.6 | 79 |
| 2002 | 54 | Male | 9.8 | 51.0 | 25.6 | 7.4 | 73 |
| 2002 | 55 | Male | 6.5 | 47.0 | 25.3 | 6.4 | 91 |
| 2002 | 56 | Male | 6.9 | 49.2 | 25.4 | 6.8 | 81 |
| 2002 | 57 | Male | 7.7 | 49.7 | 25.6 | 8.1 | 82 |
| 2002 | 58 | Male | 6.0 | 46.2 | 25.3 | 5.8 | 73 |
| 2002 | 59 | Male | 7.0 | 48.2 | 25.6 | 6.5 | 90 |
| 2002 | 60 | Male | 7.1 | 45.1 | 25.2 | 6.8 | 91 |
| 2002 | 61 | Male | 8.9 | 45.8 | 25.2 | 5.3 | 84 |
| 2002 | 62 | Male | 8.5 | 42.5 | 25.1 | 6.6 | 85 |
| 2002 | 63 | Male | 9.5 | 45.0 | 25.3 | 8.3 | 83 |
| 2002 | 64 | Male | 8.1 | 48.8 | 25.5 | 4.7 | 91 |
| 2002 | 65 | Male | 9.0 | 45.5 | 25.2 | 6.5 | 88 |
| 2002 | 66 | Male | 7.4 | 39.9 | 24.9 | 5.3 | 87 |
| 2002 | 67 | Male | 7.9 | 43.4 | 25.1 | 6.4 | 77 |
| 2002 | 68 | Male | 7.4 | 37.2 | 24.8 | 7.7 | 77 |
| 2002 | 69 | Male | 6.7 | 35.4 | 24.9 | 8.3 | 67 |
| 2002 | 70 | Male | 5.2 | 39.1 | 24.8 | 5.9 | 69 |
| 2002 | 71 | Male | 3.2 | 38.2 | 24.5 | 5.1 | 84 |
| 2002 | 72 | Male | 11.0 | 36.3 | 25.1 | 13.1 | 73 |
| 2002 | 73 | Male | 4.0 | 32.0 | 24.2 | 7.3 | 73 |
| 2002 | 74 | Male | 5.1 | 30.7 | 24.2 | 8.9 | 71 |
| 2002 | 75 | Male | 3.8 | 24.8 | 23.7 | 4.4 | 69 |
| 2002 | 76 | Male | 3.5 | 23.9 | 23.7 | 6.0 | 62 |
| 2002 | 77 | Male | 5.2 | 25.5 | 23.6 | 7.9 | 61 |
| 2002 | 78 | Male | 7.1 | 28.1 | 23.9 | 8.6 | 64 |
| 2002 | 79 | Male | 4.2 | 24.4 | 23.7 | 9.5 | 57 |
| 2002 | 80 | Male | 2.2 | 16.8 | 22.9 | 7.2 | 56 |
| 2002 | 81 | Male | 2.8 | 13.7 | 22.7 | 3.6 | 51 |
| 2002 | 82 | Male | 2.6 | 18.2 | 23.0 | 5.7 | 55 |
| 2002 | 31 | Female | 10.3 | 45.1 | 24.8 | 10.7 | 34 |
| 2002 | 32 | Female | 8.9 | 45.8 | 24.7 | 8.0 | 42 |
| 2002 | 33 | Female | 6.4 | 40.4 | 24.5 | 11.4 | 43 |
| 2002 | 34 | Female | 13.7 | 44.3 | 25.1 | 6.3 | 49 |
| 2002 | 35 | Female | 12.2 | 50.5 | 24.9 | 16.7 | 54 |
| 2002 | 36 | Female | 11.1 | 41.2 | 24.6 | 8.4 | 49 |
| 2002 | 37 | Female | 12.8 | 42.6 | 24.9 | 10.5 | 62 |
| 2002 | 38 | Female | 8.2 | 37.6 | 24.4 | 10.2 | 52 |
| 2002 | 39 | Female | 14.1 | 41.5 | 25.2 | 9.5 | 64 |
| 2002 | 40 | Female | 9.3 | 43.0 | 24.8 | 10.2 | 63 |
| 2002 | 41 | Female | 11.8 | 42.1 | 24.6 | 12.4 | 69 |
| 2002 | 42 | Female | 9.1 | 44.0 | 24.6 | 8.9 | 75 |
| 2002 | 43 | Female | 8.8 | 39.7 | 24.4 | 11.5 | 91 |
| 2002 | 44 | Female | 13.0 | 42.0 | 24.6 | 14.9 | 95 |
| 2002 | 45 | Female | 7.4 | 39.2 | 24.2 | 8.4 | 98 |
| 2002 | 46 | Female | 10.0 | 40.9 | 24.5 | 12.9 | 103 |
| 2002 | 47 | Female | 9.6 | 42.2 | 24.4 | 8.5 | 104 |
| 2002 | 48 | Female | 7.9 | 34.7 | 24.0 | 7.5 | 103 |
| 2002 | 49 | Female | 8.9 | 37.7 | 24.1 | 10.3 | 104 |
| 2002 | 50 | Female | 9.8 | 40.8 | 24.4 | 10.8 | 98 |
| 2002 | 51 | Female | 7.7 | 32.6 | 24.0 | 8.7 | 89 |
| 2002 | 52 | Female | 8.2 | 34.1 | 23.8 | 7.9 | 100 |
| 2002 | 53 | Female | 10.0 | 35.7 | 23.9 | 9.0 | 63 |
| 2002 | 54 | Female | 8.5 | 28.5 | 23.6 | 13.2 | 67 |
| 2002 | 55 | Female | 7.6 | 32.0 | 23.6 | 11.1 | 76 |
| 2002 | 56 | Female | 9.0 | 35.4 | 24.0 | 7.7 | 81 |
| 2002 | 57 | Female | 7.5 | 26.9 | 23.1 | 11.4 | 91 |
| 2002 | 58 | Female | 9.3 | 32.6 | 23.9 | 11.3 | 101 |
| 2002 | 59 | Female | 9.1 | 30.3 | 23.6 | 9.4 | 94 |
| 2002 | 60 | Female | 10.2 | 27.8 | 23.4 | 9.9 | 112 |
| 2002 | 61 | Female | 6.5 | 27.0 | 23.2 | 12.2 | 106 |
| 2002 | 62 | Female | 6.8 | 29.2 | 23.5 | 7.9 | 110 |
| 2002 | 63 | Female | 9.6 | 29.3 | 23.8 | 10.8 | 112 |
| 2002 | 64 | Female | 7.4 | 31.0 | 23.6 | 12.0 | 124 |
| 2002 | 65 | Female | 7.9 | 27.3 | 23.3 | 7.3 | 109 |
| 2002 | 66 | Female | 7.5 | 28.4 | 23.4 | 12.0 | 120 |
| 2002 | 67 | Female | 8.1 | 30.0 | 23.4 | 9.5 | 110 |
| 2002 | 68 | Female | 6.3 | 22.0 | 22.8 | 9.3 | 106 |
| 2002 | 69 | Female | 7.9 | 29.3 | 23.4 | 10.6 | 92 |
| 2002 | 70 | Female | 8.2 | 26.6 | 23.1 | 14.4 | 83 |
| 2002 | 71 | Female | 4.7 | 22.0 | 23.0 | 8.7 | 97 |
| 2002 | 72 | Female | 5.2 | 22.7 | 23.2 | 9.1 | 96 |
| 2002 | 73 | Female | 4.2 | 17.4 | 22.4 | 8.8 | 92 |
| 2002 | 74 | Female | 7.5 | 24.5 | 23.2 | 12.1 | 93 |
| 2002 | 75 | Female | 3.8 | 20.6 | 22.6 | 6.7 | 91 |
| 2002 | 76 | Female | 3.9 | 19.0 | 22.6 | 7.5 | 75 |
| 2002 | 77 | Female | 3.6 | 18.5 | 22.4 | 7.8 | 80 |
| 2002 | 78 | Female | 4.0 | 15.1 | 22.1 | 9.9 | 79 |
| 2002 | 79 | Female | 4.2 | 15.4 | 22.2 | 11.3 | 76 |
| 2002 | 80 | Female | 1.5 | 10.3 | 21.8 | 6.4 | 79 |
| 2002 | 81 | Female | 1.3 | 13.9 | 21.8 | 8.1 | 58 |
| 2002 | 82 | Female | 3.2 | 14.3 | 21.7 | 11.3 | 63 |
| 1994 | 53 | Male | 6.5 | 42.4 | 24.7 | 6.7 | 108 |
| 1994 | 54 | Male | 5.5 | 39.8 | 24.6 | 6.9 | 97 |
| 1994 | 55 | Male | 3.5 | 36.3 | 24.2 | 4.9 | 119 |
| 1994 | 56 | Male | 5.8 | 37.0 | 24.4 | 4.6 | 109 |
| 1994 | 57 | Male | 3.4 | 33.4 | 24.2 | 4.0 | 115 |
| 1994 | 58 | Male | 3.2 | 35.3 | 24.2 | 3.5 | 112 |
| 1994 | 59 | Male | 3.1 | 33.9 | 24.2 | 5.2 | 132 |
| 1994 | 60 | Male | 3.3 | 33.2 | 24.1 | 4.6 | 132 |
| 1994 | 61 | Male | 4.2 | 34.1 | 24.2 | 5.1 | 123 |
| 1994 | 62 | Male | 3.9 | 33.1 | 24.1 | 5.3 | 131 |
| 1994 | 63 | Male | 4.0 | 33.1 | 24.1 | 6.3 | 121 |
| 1994 | 64 | Male | 3.1 | 35.2 | 24.1 | 6.3 | 133 |
| 1994 | 65 | Male | 3.5 | 26.9 | 23.7 | 4.3 | 140 |
| 1994 | 66 | Male | 1.6 | 25.5 | 23.5 | 6.2 | 136 |
| 1994 | 67 | Male | 3.6 | 26.4 | 23.7 | 6.6 | 120 |
| 1994 | 68 | Male | 3.4 | 23.5 | 23.4 | 6.3 | 116 |
| 1994 | 69 | Male | 4.0 | 19.8 | 23.4 | 8.1 | 109 |
| 1994 | 70 | Male | 2.9 | 17.3 | 23.0 | 6.3 | 100 |
| 1994 | 71 | Male | 1.0 | 15.7 | 22.9 | 4.0 | 124 |
| 1994 | 72 | Male | 3.6 | 19.7 | 23.0 | 7.8 | 108 |
| 1994 | 73 | Male | 0.5 | 11.7 | 22.4 | 4.0 | 106 |
| 1994 | 74 | Male | 0.5 | 13.1 | 22.2 | 3.9 | 102 |
| 1994 | 75 | Male | 1.6 | 8.8 | 22.0 | 5.5 | 105 |
| 1994 | 76 | Male | 0.2 | 6.6 | 21.4 | 3.6 | 99 |
| 1994 | 77 | Male | 0.7 | 9.3 | 21.0 | 6.1 | 100 |
| 1994 | 78 | Male | 0.2 | 6.5 | 20.1 | 4.4 | 100 |
| 1994 | 79 | Male | 1.5 | 6.8 | 19.8 | 7.2 | 94 |
| 1994 | 80 | Male | 0.5 | 5.2 | 18.8 | 4.6 | 90 |
| 1994 | 81 | Male | 0.3 | 7.5 | 18.1 | 4.3 | 79 |
| 1994 | 82 | Male | 0.5 | 7.6 | 17.8 | 4.5 | 82 |
| 1994 | 53 | Female | 5.5 | 19.5 | 22.7 | 7.3 | 79 |
| 1994 | 54 | Female | 5.8 | 17.1 | 22.6 | 14.1 | 84 |
| 1994 | 55 | Female | 5.3 | 18.4 | 22.5 | 6.0 | 96 |
| 1994 | 56 | Female | 3.7 | 17.2 | 22.5 | 9.5 | 98 |
| 1994 | 57 | Female | 3.3 | 18.0 | 22.3 | 9.3 | 112 |
| 1994 | 58 | Female | 5.0 | 19.6 | 22.4 | 9.8 | 130 |
| 1994 | 59 | Female | 4.1 | 17.9 | 22.3 | 10.1 | 125 |
| 1994 | 60 | Female | 5.4 | 19.2 | 22.4 | 9.3 | 144 |
| 1994 | 61 | Female | 3.2 | 18.4 | 22.1 | 5.9 | 142 |
| 1994 | 62 | Female | 3.8 | 17.1 | 22.3 | 8.0 | 147 |
| 1994 | 63 | Female | 6.0 | 19.3 | 22.5 | 8.5 | 153 |
| 1994 | 64 | Female | 4.5 | 18.0 | 22.4 | 8.7 | 167 |
| 1994 | 65 | Female | 3.9 | 16.5 | 22.3 | 7.1 | 154 |
| 1994 | 66 | Female | 2.3 | 17.0 | 22.2 | 5.8 | 161 |
| 1994 | 67 | Female | 5.6 | 18.9 | 22.3 | 10.2 | 146 |
| 1994 | 68 | Female | 3.7 | 13.5 | 21.8 | 7.6 | 143 |
| 1994 | 69 | Female | 3.2 | 15.5 | 22.0 | 7.5 | 122 |
| 1994 | 70 | Female | 3.4 | 13.2 | 21.8 | 10.6 | 113 |
| 1994 | 71 | Female | 2.1 | 12.0 | 21.7 | 7.1 | 129 |
| 1994 | 72 | Female | 2.8 | 10.2 | 21.6 | 7.1 | 121 |
| 1994 | 73 | Female | 1.4 | 10.0 | 21.4 | 6.8 | 122 |
| 1994 | 74 | Female | 1.8 | 10.4 | 21.4 | 7.9 | 118 |
| 1994 | 75 | Female | 1.3 | 6.8 | 20.9 | 5.6 | 118 |
| 1994 | 76 | Female | 1.2 | 6.1 | 20.9 | 4.6 | 99 |
| 1994 | 77 | Female | 0.5 | 3.6 | 20.5 | 4.4 | 102 |
| 1994 | 78 | Female | 1.2 | 4.9 | 20.3 | 6.4 | 103 |
| 1994 | 79 | Female | 1.5 | 4.4 | 19.8 | 7.5 | 99 |
| 1994 | 80 | Female | 0.3 | 2.3 | 18.9 | 4.5 | 93 |
| 1994 | 81 | Female | 0.0 | 3.4 | 18.2 | 3.8 | 81 |
| 1994 | 82 | Female | 0.9 | 7.8 | 18.1 | 5.6 | 72 |
